# The non-indigenous *Oithona davisae* in a Mediterranean transitional environment: coexistence patterns with competing species

**DOI:** 10.1038/s41598-021-87662-5

**Published:** 2021-04-16

**Authors:** Marco Pansera, Elisa Camatti, Anna Schroeder, Giacomo Zagami, Alessandro Bergamasco

**Affiliations:** 1 Institute of Marine Sciences, National Research Council, Arsenale Tesa 104, Castello 2737/F, 30122 Venice, Italy; 2 Faculty of Environmental Life Sciences, University of Trieste, Via Licio Giorgieri 5, 34127 Trieste, Italy; 3 Department of Chemical, Biological, Pharmaceutical and Environmental Sciences, University of Messina, Viale F. Stagno d’Alcontres, 31, S. Agata, 98166 Messina, Italy

**Keywords:** Ecology, Environmental sciences

## Abstract

The Venice lagoon (VL) has been recognized as a hot spot of introduction of non-indigenous species (NIS), due to several anthropogenic factors and environmental stressors that combined may facilitate NIS invasions. In the last decades an increasing number of zooplankton NIS have been observed in the VL. This work aims to provide a picture of the annual cycle and distribution of the recently recorded non-indigenous copepod *Oithona davisae,* considering the coexistence patterns with the congeneric resident *Oithona nana*. Therefore, zooplankton samplings were carried out monthly from August 2016 to July 2017 at five Long-Term Ecological Research LTER stations in the VL. *Oithona davisae* showed a persistent occurrence throughout the year with the highest abundances in the warm season and in the inner areas, while the congeneric *O. nana*, showing a different distribution pattern, resulted more abundant near the inlets of the Lagoon, where *O. davisae* reached the minimum density. *Oithona davisae* seems to find local conditions that promote its settlement and distribution, especially in the inner and more trophic lagoon sites. In other European coastal embayments or transitional waters, *O. davisae* occupied the niche left by the indigenous *O. nana* or can replace this congeneric species through competitive exclusion mechanisms. Our data indicate that, for now, such species replacement has not occurred in the VL. One of the causes is the extreme variety of habitats and niches offered by this environment allowing a balanced coexistence with *O. nana* and in general with the resident copepod community.

## Introduction

Alien species, also called non-native or non-indigenous species (NIS), are defined as those species that are introduced into an area beyond their natural geographic range. The effects on the environment of some invasive non-indigenous species have been recognized as one of the major threats to the conservation of biodiversity and functioning of marine ecosystems^1–3^. The introduction of NIS is continuously increasing, sometimes with negative consequences for the local food web or for ecosystem services^4^ [and references therein]. For this, control and mitigation strategies are increasingly demanded by stakeholders. In recent years, the issue of alien species has been addressed in the EU Marine Strategy Framework Directive. Non-indigenous aquatic species are of primary concern to many regulating authorities and are seen as one of the top four anthropogenic threats of the worlds’ oceans^5^.

**Figure 1:**
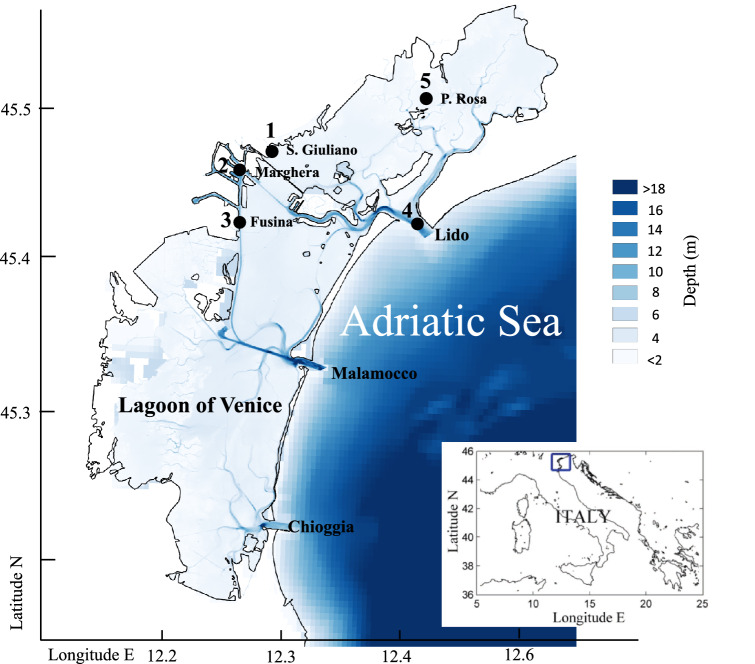
Study area (Lagoon of Venice, Italy) and location of sampling stations, modified from^36^.

The Mediterranean Sea is one of the most affected regions with increasing numbers of NIS due to accidental synanthropic introductions; among the reported 821 alien species by the year 2016, 613 are considered established, and 42 are planktonic copepods^6,7^. In the last decades, an increasing number of invasive zooplankton species, like the copepods *Acartia tonsa*, *Pseudodiaptomus marinus, Oithona davisae* and the lobate ctenophore *Mnemiopsis leidyi*, have become established or been observed in Venice Lagoon (VL)^8–10^, a microtidal Mediterranean environment located in the North Adriatic Sea (Fig. 1). Currently, the VL is considered a main hotspot of non-indigenous species introductions in the Italian coasts^11^ due to its ecological features and the pressures by anthropic activities. The VL, affected by intense maritime traffic, presents a high risk of NIS introduction through ballast water, a key global vector in human-mediated invasions, acting as a fast dispersal mechanism for many marine taxa^9,11^.

The key position of zooplankton within the aquatic trophic network promoted the interest in studies related to the impact of the introduction of new zooplanktonic species, especially of those related to commercial activities such as fishing or aquaculture. Copepods represent the most abundant group within the zooplankton community and the food source of most commercially exploited fish. In particular, within copepods, the ubiquitous genus *Oithona* is considered the most abundant group in the oceans^12^, playing an important role in food webs as a food source for planktivorous fishes and fish larvae^13,14^. Oithonids are a highly diverse group that can be found in a variety of environments from the open ocean to transitional waters and marine coastal lakes^15^.

*Oithona davisae* Ferrari & Orsi, 1984 (Copepoda: cyclopoida) is a nearshore and estuarine species, indigenous to the temperate coastal waters of the Indo West-Pacific^16^, inhabiting eutrophic bays and transitional waters^17,18^. Although *O. davisae* is a thermophilic species, it has been found in areas with temperatures ranging from below 0 to 29 °C^19,20^. In the last forty years, *O. davisae* has spread to the Pacific Ocean, the Atlantic Ocean and the Black Sea^16,21–26^. Until now, in the Mediterranean Sea, *O. davisae* has been found in limited areas as inner parts of the enclosed bay of Barcelona (Spain)^13^, the Izmir Bay in the Aegean Sea (Turkey)^27^, the coastal lakes Faro and Ganzirri of Messina (Italy)^18^ and recently, in the VL where it was firstly recorded in 2014^9^.

The niche contender *O. davisae* can have a strong impact on the ecosystem structure and functioning due to its ability to form dense populations in short times^17^. In the VL, the role of this species within the local copepod community remains still to be clarified.

The aim of the present work is to provide a picture of the spatial and temporal distribution of *O. davisae* in the VL. Moreover, the niche relationships between this species and the copepod community have been analysed, focusing in particular on the possible competition with the resident congeneric *Oithona nana.* For this, different areas of the lagoon have been chosen, in relation to a gradient of environmental parameters. Finally, niche apportionment models have been applied to investigate possible mechanisms regulating the resource allocation within the copepod community and to identify mechanisms that could influence the distribution of *O. davisae* in different lagoon habitat.

## Methods

### Study area

The Venice Lagoon is one of the 25 research sites belonging to the Italian Long-Term Ecological Research Network (LTER-Italy, www.lteritalia.it). It is a shallow coastal water body located in the north-western part of the Adriatic Sea, with a surface area of about 500 km^2^ (Fig. 1) and an average depth of 1.5 m. It is connected with the Adriatic Sea through three inlets allowing water and sediment exchange with the sea, named Lido, Malamocco and Chioggia. The semi-diurnal microtidal regime of the VL has with a mean range of 0.40 m during neap tides and about 0.80 m during spring tides. The residence times ranged from few days, in the proximity of the three inlets, to over 60 days in the inner lagoon areas^28^, while the amount of seawater that is exchanged during each tidal cycle is about one third of the total volume of the lagoon^29^. The brackish nature of the VL results from the freshwater inputs from the drainage basin, which is about 35 m^3^ s^−1^^30^. The lagoon is a heterogeneous and complex system, characterized by different physical and environmental gradients, and a mosaic of landforms and habitats that are the result of natural and man-induced drivers^31^; in the entire lagoon basin, typical salinity and temperature range of 9–37 and 5–28 °C, respectively^32,33^. The VL is affected by several anthropogenic pressures like fisheries, shellfish culture, domestic and industrial discharges and maritime transport^34,35^.

From a biological point of view, planktonic copepods represent the dominant fraction of zooplankton assemblages across the VL, albeit with varying species composition along the salinity gradient^37^. In the VL, resident zooplankton species are particularly able to adapt to highly variable environmental conditions. In the inner areas, characterized by salt marshes and channels, the copepod *A. tonsa*, a typical species in transitional waters, is dominant in the warm period^36^. Towards the inlets, with the increasing of marine features, neritic and marine species such as *Acartia clausi*, *Paracalanus parvus* and *Centropages spp.* begin to dominate^38^. In some periods, especially during the warm season and in areas close to the inlets, the presence of cladocerans is relevant (*Evadne spp., Podon spp.* and *Penilia avirostris*), while in the internal lagoon basin, meroplanktonic forms (such as larval stages of Decapoda, Bivalvia and Polychaeta) represent an important fraction of the mesozooplankton community^37,38^. Within oithonids, only *O. nana* represents a dominant component of the summer zooplankton lagoon assemblage, while *O. similis* and *O. plumifera*, the other two oithonids detected in the Lagoon, were generally found in areas with more marine characteristics and always with low abundances^39^. Zooplankton sampling was carried out in the context of the LTER monitoring activities, which studied since few decades five stations located in the central and the northern part of the Venice Lagoon (Fig. 1) and representing a gradient of environmental conditions and different anthropogenic impacts. Station St.1 (S. Giuliano) is affected by urban discharges, St.2 (Marghera) is located in the industrial port area influenced by industrial pollutants and St.3 (Fusina) receives warm water from a thermoelectric power plant. These three stations, considered the inners stations, are characterized by high water residence times of 18–25 days^28^. The inlet station St.4 (Lido), located in the northernmost inlet of the Lagoon, is influenced by Adriatic coastal waters with a residence time of few hours and finally, St.5 (Palude della Rosa) is a typical lagoon environment influenced both by fresh water and by sea water inputs and characterized by a residence time of 6–12 days^28^.

### Sampling methods and laboratory analysis

To study *O. davisae* and *O. nana*, quantitative samples were collected monthly, from August 2016 to July 2017, by horizontal hauls with the HydroBios Apstein net (mouth diameter 0.17 m, mesh size 80 μm). The 80 μm Apstein net was operated by hand from the boat by towing 15 m rope length at 0.3 m s^−1^ approximately, resulting a filtered volume of 0.34 m^31^. This particularly small mesh size was adopted, as it is known that both *O. davisae* and *O. nana*, being relatively small species, may pass through the classically used nets with a 200 μm mesh^40^. The characterization of the rest of the mesozooplankton community was based instead on seasonal samplings, from May 2014 to February 2017 (Supplementary Table S4), performed by the ongoing long-term zooplankton monitoring activities. Those zooplankton community samples were collected by horizontal hauls of HydroBios Apstein net (mouth diameter 0.4 m, mesh size 200 μm) and of WP2 standard net (mouth diameter 0,57 m, mesh size 200 µm) equipped with a flowmeter HydroBios to calculate the filtered water volumes (7 to 50 m^3^).

A multiparametric CTD probe (SBE 19plus) was used to measure environmental parameters like temperature, salinity, oxygen and chlorophyll *a* at the sampling sites, while the water transparency was measured with the Secchi disk. The zooplankton samples were fixed in a solution of 4% sodium tetraborate buffered formalin and ambient water. In the laboratory, copepods were identified and counted, according to the International Council for the Exploration of the Sea (ICES) protocol^41^ to species level, where possible, or to higher taxonomic levels. As males of the genus *Oithona* could not be easily identified at species level, only copepodites and females were considered regarding the two species *O. davisae* and *O. nana*. While adult males and earlier stages (nauplii) were not counted.

### Statistical analysis/data processing

Spatial and temporal patterns of the environmental factors based on Euclidean distances were assessed using repeated-measure permutational analysis of variance (PERMANOVA) with the sampling months as fixed factor and the stations as random factor (PRIMER 6 + and PERMANOVA software package; PRIMER-E, Ltd., UK). In order to visualize the similarities between the samples in terms of environmental conditions, a PCoA (Principal coordinates analysis) based on Euclidean distances and the vectors of environmental parameters was performed, and the occurrence of the two oithonids, *O. nana* and *O. davisae,* (as Bray–Curtis-Similarity matrix) was superimposed. Moreover, the Spearman correlation of the environmental and community similarity matrices were calculated with the software package PRIMER6+, utilising the package RELATE^42^ in order to estimate the relationship between the environmental factors and the abundances of the two *Oithona* species.

The Pearson correlations to estimate the relationship between the abundances of the two *Oithona* species and the environmental parameters were calculated with the Software package Statgraphics^43^. To detect differences between and within groups of abundance data (months, stations), ANOVA LSD test was employed after the checking for the homogeneity of the variances.

### Rank abundance distributions—RAD

The characterisation of abundance and distribution of most *Oithona* species calls for the use of sampling nets with a mesh size smaller than 200 μm^12,40^. Parallel comparison of efficiency of different sampling devices in catching *Oithona* species has been only seldom faced and investigated^15^ and no conclusive guidelines have been issued to scale the catches or merge the data collected with different approaches in single tows.

Nevertheless, for the scope of the present study, it was necessary to detail the presence/absence and relative abundances of to the two species (*O. davisae* and *O. nana*) within the picture of the overall copepod community in each station and possibly in each seasonal/environmental condition.

Given the quite even distribution of the samplings events during the year (monthly with the 80 μm net, and almost seasonally during 2014–2017 period with the 200 μm net), we approached the issue by assuming as “typical” the yearly trend of *Oithona* species coming from the samplings with 80 μm net (Supplementary Fig. S1). The linear interpolation of the expected abundances in specific calendar days made possible their merging with the specimen counts of the 200 μm samplings.

In this way we were able to construct the synthetic Rank Abundance Distributions (RAD) of the overall copepod community in each station by adding together all the catches collected with both nets (80 μm and 200 μm) and then calculating the relative abundances and ranks of every species. In particular we obtained, for each station, a pool of copepod communities that (1) integrates the species abundances of standard 200 μm net and the 80 μm net counts for *O. nana* and *O. davisae*, and (2) corresponds to several seasonal conditions during the year. These pools were also used to investigate the mechanisms of niche apportionment.

### Niche apportionment models

To give insights into the mechanisms driving the structuration of the community, captured by the relative abundance distributions of species, the Niche Apportionment Models (NAM) were used.

The theoretical framework regarding the NAMs has been developed in early 90 s by Tokeshi^44^ who built on Sugihara’s niche-hierarchy models (1980), to support in the practical problems of fitting stochastic models to real data for the interpretation of species abundance patterns. The developed framework gives logical coherence to a range of niche apportionment models that can be categorized through the sequential breakage process of total niche. To describe how species break up the resource pool, determining the distribution of abundance of different species^44,45^, the theory proposed by Tokeshi ^45–47^ takes into consideration six different models which are (sorted by decreasing evenness): Dominance Preemption (DP), Random Assortment (RA), Random Fraction (RF), Power Fraction (PF), MacArthur Fraction (MF) and Dominance Decay (DD); the abundance of a species is assumed to directly correspond to the amount of niche/resource apportioned to that species.

In these models of sequential breakage of the total niche, the dominance pattern depends on the probability p associated to each niche fraction. The resulting Species Abundance Distribution (SAD) vary from uneven patterns like the geometric series to extremely even ones similar to the broken-stick model^48^.

The power of this analysis ultimately relies on the robustness of the Species Abundance Distribution within a community in catching (in the shape of the SAD itself) the underlying mechanisms that drive the relations (coexistence, competition, etc.) among the species and therefore the structuration of the community. So that fitting one of the proposed stochastic models to the SAD (i.e. to find the model that fits better than others to the survey data, and this can be done with classical statistical methods) means that the probability of that specific sequential niche breakage (caught by the modelled scheme) is higher than others.

In order to highlight the mechanisms regulating the resource allocation within a community of related species, several authors suggest applying the NAMs only on that specific group of taxonomically related species or trophic guild^45–47^. As copepods represent about 80% of the total zooplankton community in the VL^38^, we applied the NAMs to this dominant planktonic group in order to elucidate the mechanisms regulating the resource allocation in different areas and to understand if those mechanisms could explain the different distribution of *O. davisae* and *O. nana*. This is the first attempt to apply this approach to copepods communities. In fact, to date, these models have been used to explain and describe changes in relative abundance distributions of terrestrial and aquatic communities of both fresh and marine waters, like larval chironomid, stream fish assemblages, salt meadow vegetation, dragonfly community, terrestrial arthropods, marine macroalgae and phytoplankton community^44,46,49–51^. The analyses have been performed using the R-package ‘nicheApport’ (available in GitHub, 2017).

## Results

### Environmental characteristics

**Figure 2:**
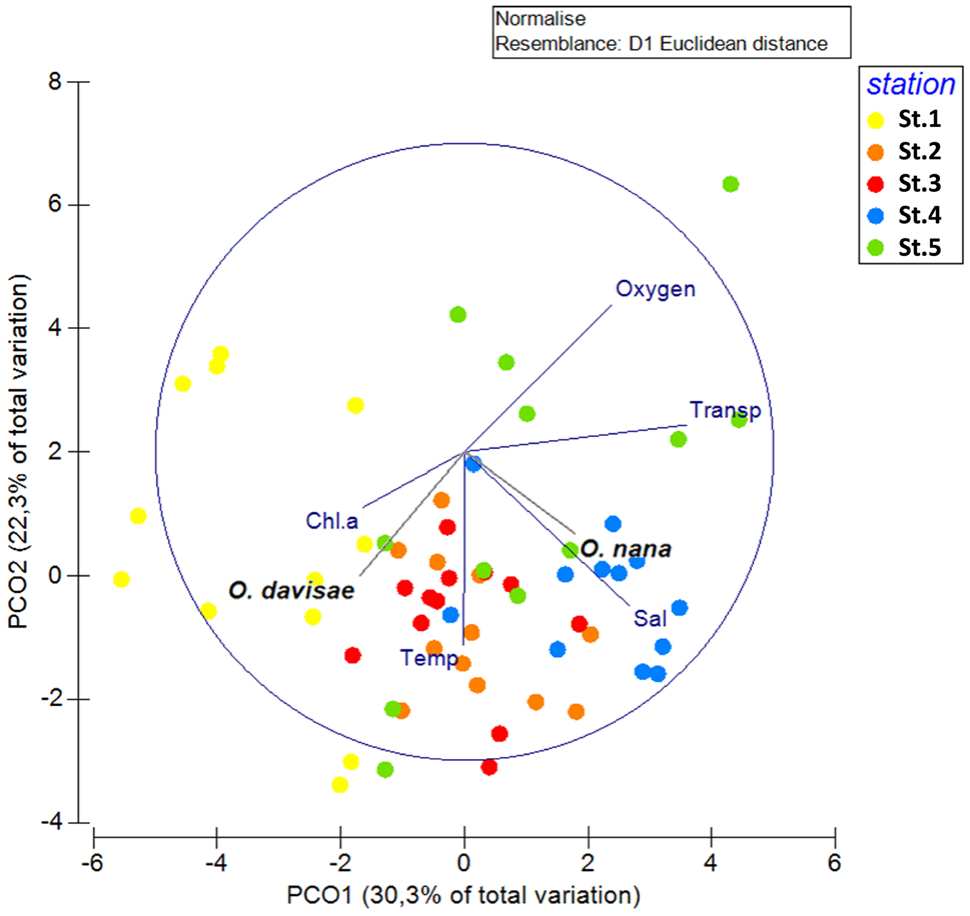
Principal coordinates analysis of environmental parameters in the five stations overlaying the correlation with the abundance of the two Oithonidae.

The investigated area is characterized by a high variability of abiotic conditions typical of transitional environments. The environmental parameters differ significantly both spatially, between stations, as well as temporally, between the month of sampling (PERMANOVA Table 1). During summer, higher temperatures and Chl-*a* values and lower transparency were measured compared to other seasons. The salinity, influenced by fresh and marine water inputs, varies between stations and throughout the year. Trophic and salinity gradients are evident with the highest mean Chl-*a* values at the inner stations and the highest mean salinity value in the area close to the inlet (St. 4) (Table 2, Fig. 2).

**Table 1:** Spatial and temporal patterns of the environmental factors based on Euclidean distances assessed using repeated-measure permutational analysis of variance (PERMANOVA) with the sampling months as fixed factor and the stations as random factor.

Source	df	SS	MS	Pseudo-F	P (perm)	Unique perms
Station	4	83.859	20.965	8.9721	0.001	999
Month	11	108.33	9.848	4.2145	0.001	994
Res	44	102.81	2.3367			
Total	59	295

**Table 2:** Minimum, maximum, mean and standard deviation of the temperature, salinity, oxygen, chlorophyll *a* and Secchi disk values measured at lagoon stations and Pearson correlations values for the two Oithonidae and the environmental parameters.

Station	Temperature [°C]				Salinity				Oxygen [%]				Chl-*a* [μg l^−1^]				Secchi disk [m]			
	Min	Max	Mean	SD	Min	Max	Mean	SD	Min	Max	Mean	SD	Min	Max	Mean	SD	Min	Max	Mean	SD
1	1.2	28.2	17.6	8.3	21.0	30.5	26.2	3.0	96.6	131.2	111.3	10.1	0.44	41.42	6.31	11.89	0.3	0.9	0.6	0.2
2	5.6	30.0	18.6	7.8	29.8	32.7	31.4	0.8	91.6	133.9	106.5	14.3	0.10	7.15	1.90	2.42	1.0	2.3	1.6	0.4
3	7.7	31.6	22.4	6,8	22.6	32.9	30.5	2.6	102.6	126.6	112.4	9.3	0.21	4.26	1.31	1.38	0.7	2.8	1.2	0.6
4	5.0	27.3	17.0	6.9	29.5	35.3	33.1	1.7	99.9	142.9	114.7	13.2	0.56	2.34	1.27	0.57	0.6	4.2	2.7	1.1
5	1.2	28.7	17.3	8.1	23.4	32.5	28.1	2.7	68.7	203.5	134.4	40.3	0.36	6.98	1.89	1.96	0.4	2.7	1.4	0.8
*O. davisae*	r = 0.255 *p* = 0.051				r = − 0.104 *p* = 0.434				r = − 0.029 *p* = 0.82				r = 0.358 *p* = 0.005 *				r = − 0.200 *p* = 0.130			
*O. nana*	r = 0.212 *p* = 0.106				r = 0.295 *p* = 0.023 *				r = 0.060 *p* = 0.654				r = − 0.062 *p* = 0.640				r = 0.430 *p* = 0.001 *

Station St.1 is characterized by the highest Chl-*a* values (41.42 µg/L), low transparency (mean: 0.6 ± 0.2 m) and low salinity (mean: 26.18 ± 3.03) compared to the other stations. Also, at St.1 the minimum temperature values are the lowest (1.2 °C a January 2017). Station St.2, with a moderate oversaturation in oxygen compared to the other stations (106.49% ± 14.3), showes intermediate ranges regarding all other parameters, characterizing it as an intermediate station. Station St.3, influenced by a thermoelectric power plant, showes relatively high temperatures (min. 7.7 °C, max. 31.6 °C). Station St.4, the most influenced by the sea, exhibites highest salinity values (mean: 33.1 ± 1.71 °C; max 35.3), lower temperature maxima compared to the lagoon stations and the highest transparency (mean: 2.75 m; max: 4.2 m). Finally, St.5 is characterized by a high variability regarding environmental parameters and particularly by the most varying oxygen values, ranging from 68.73% to 203.51%. Environmental parameters for each station throughout the study period can be found in Table 2.

Hence, St.1 and 4, are characterized by higher trophic condition coupled with high residence times^28^ at St.1 and higher salinity and transparency and lower Chl-*a* values at the inlet station St.4.

### Spatial and temporal distribution

**Figure 3:**
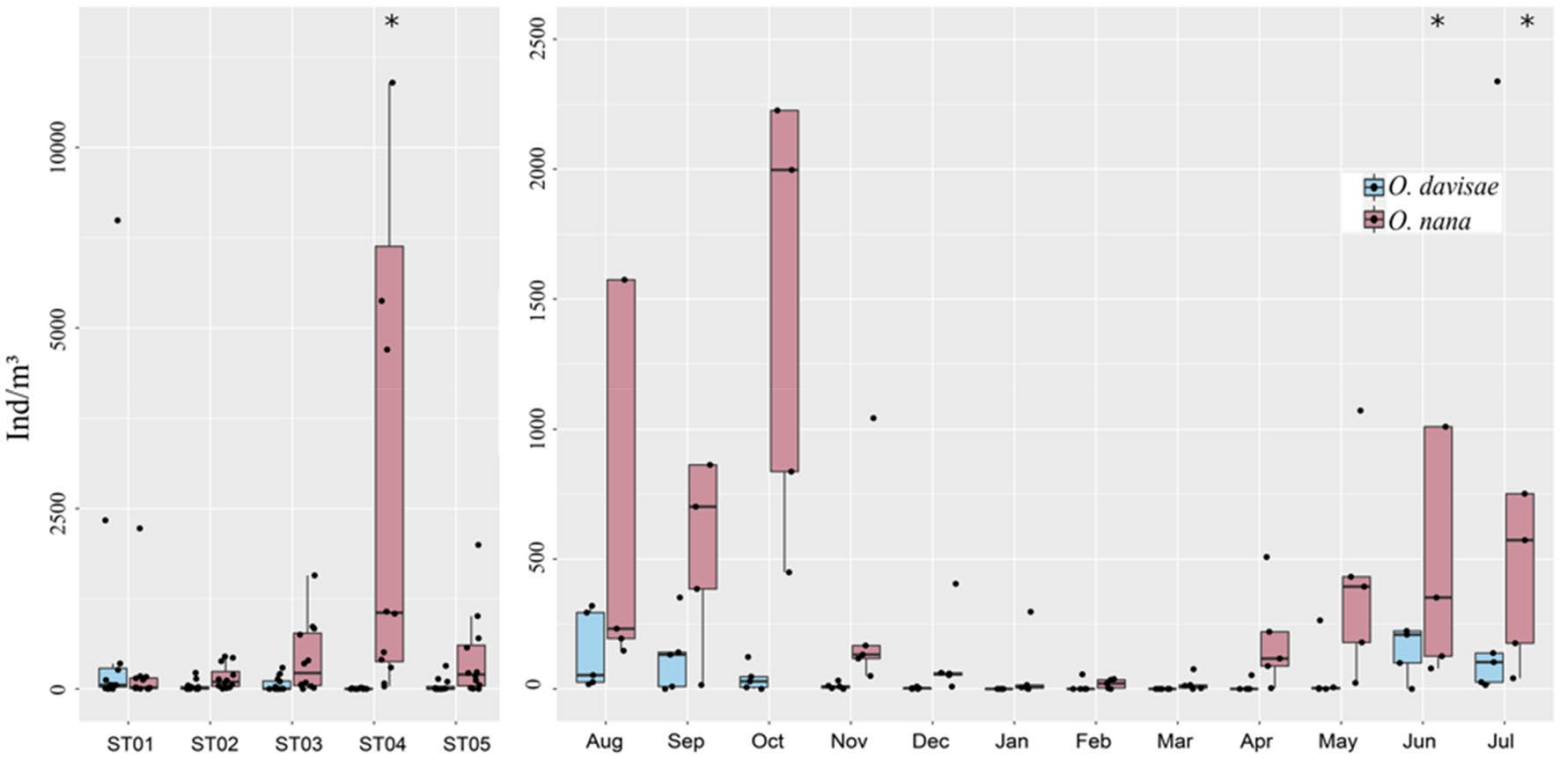
Boxplot representing the abundances of the two oithonids, *Oithona davisae* and *Oithona nana* grouped by seasons and by stations. Black dots mark the abundance values, while asterisks mark the outliers.

**Figure 4:**
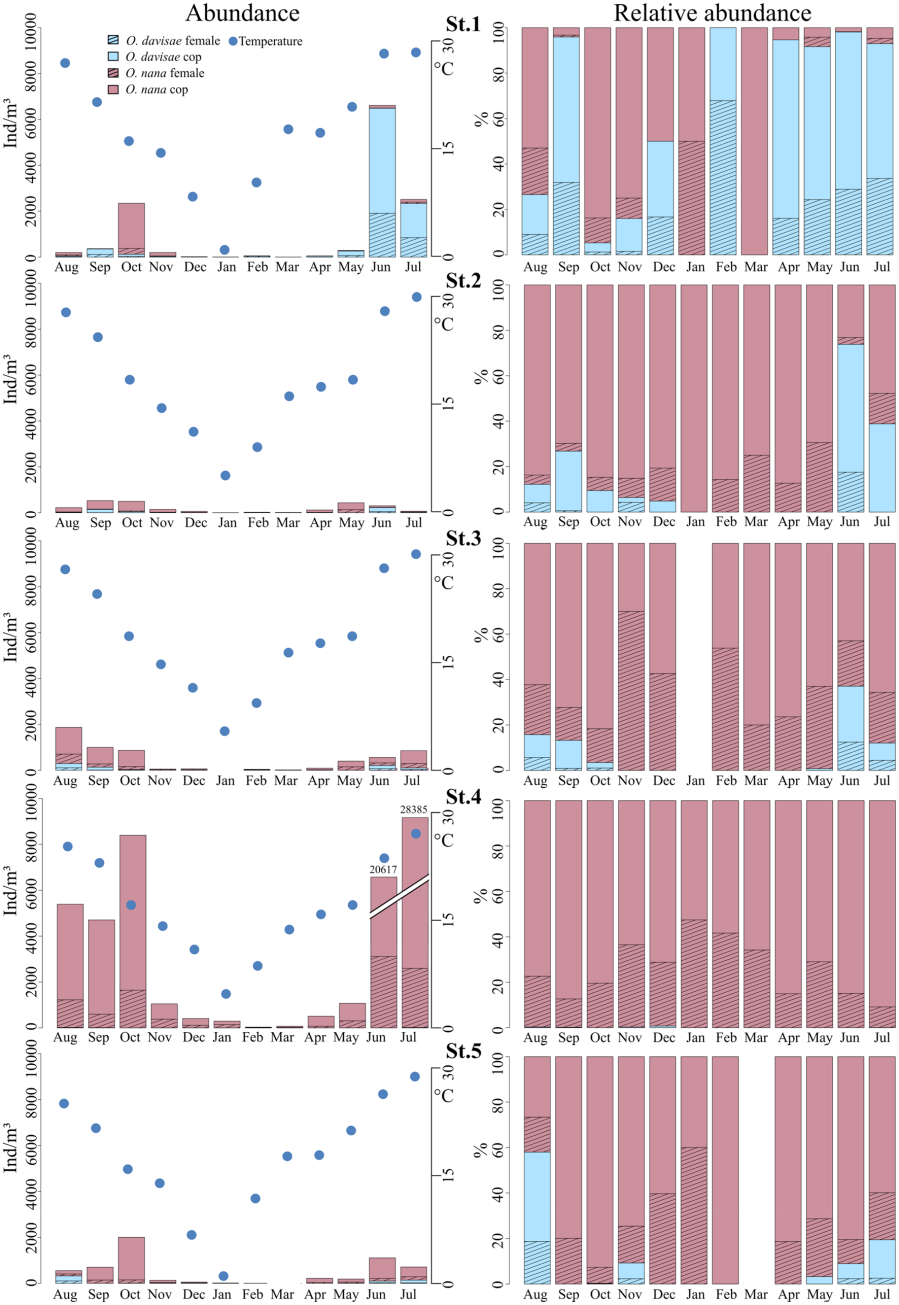
Absolute and relative abundance of copepodites and females of *Oithona davisae* and *Oithona nana* and temperature trends by station and month.

*Oithona davisae* was found to be present in all investigated areas, with significantly higher abundances at the inner station (St.1) (max. 6,490 ind/m^3^, June) compared to the other stations (*p* < 0.05, Fisher LSD test) where the densities never exceeded 320 ind/m^3^ (August at St.5) (Figs. 3, 4). At St.4 the abundances were always very low (max 18 ind/m^3^, August). In contrast, the congeneric *O. nana* showed a opposite distribution pattern as it was significantly more abundant at St.4 than the other stations (*p* < 0.05, Fisher LSD test) (max. 28,370 ind/m^3^, July). Regarding the temporal distribution, *O. davisae* showed generally the highest abundances in the warm season (from June to October), while the species was almost absent at all stations from January to April except in February and April at St. 1, although with a density of just over 50 ind/m^3^ (Fig. 4). In fact, O. davisae was significantly more abundant in June (*p* < 0.05, Fisher LSD test) than in the rest of year (except for July) reaching densities of 6,490 ind/m^3^. Similarly, *O. nana* showed its maximal abundances in June and July with 20,617 and 28,370 ind/m^3^, respectively (Figs. 3, 4), but differences to the other months were not statistically significant. In the cold period, from December to April, *O. nana* showed low abundances and it did not exceed the 508 ind/m^3^ detected in April at St.4. This species resulted absent at St.1 in February, at St.3 in January and at St.5 in March (Fig. 4).

*O. nana* generally presented abundances higher than *O. davisae* in about 87% of the samples. Moreover, in 65% of the samples its relative abundance, within these two oithonids, was greater than 90%; while *O. davisae* reached relative abundances of more than 90% only at St.1 in February, from April to July and in September (Fig. 4).

After one year of observation, it emerged that the two species seem to be distributed along opposite physico-chemical gradients. While *O. nana* resulted to be more abundant near the inlets, where *O. davisae* reaches its minimum, *O. davisae* is more abundant in the inner areas, where *O. nana* occurs with low abundances (Fig. 4). When calculating the Pearson correlation of the two species to every single parameter, it is evident, that the presence of *O. davisae* is significantly positively correlated to Chl-*a* (r = 0.358, *p* = 0.005), while *O. nana* is weakly though significantly positively correlated to salinity (r = 0.295, *p* = 0.0231) and significantly to transparency (r = 0.43, *p* = 0.0007) (Table 1, Fig. 2). In general, our data show weak correlations with all the environmental parameters considered, except for chlorophyll *a* in which the correlation is medium and significant; with respect to temperature, the correlation is at the limits of significance (for *O. davisae*) and there is a positive correlation signal which supports the seasonal characteristics of the species. The correlation with chlorophyll is however a clear signal of a larger scale trophic dynamics, which supports a trophic network, also of prey and predators, within which *O. davisae* is placed.

### Synthetic rank abundance distributions

Species RADs (Synthetic Rank Abundance Distributions) were used to highlight relative rank abundance and potential dominance of *O. davisae* within the copepod community at different seasons and areas of the lagoon, comparing it especially to the congeneric *O. nana*. As St.1 and St.4 were those stations where the two species reached their maxima in abundance, they were selected for this investigation.

**Figure 5:**
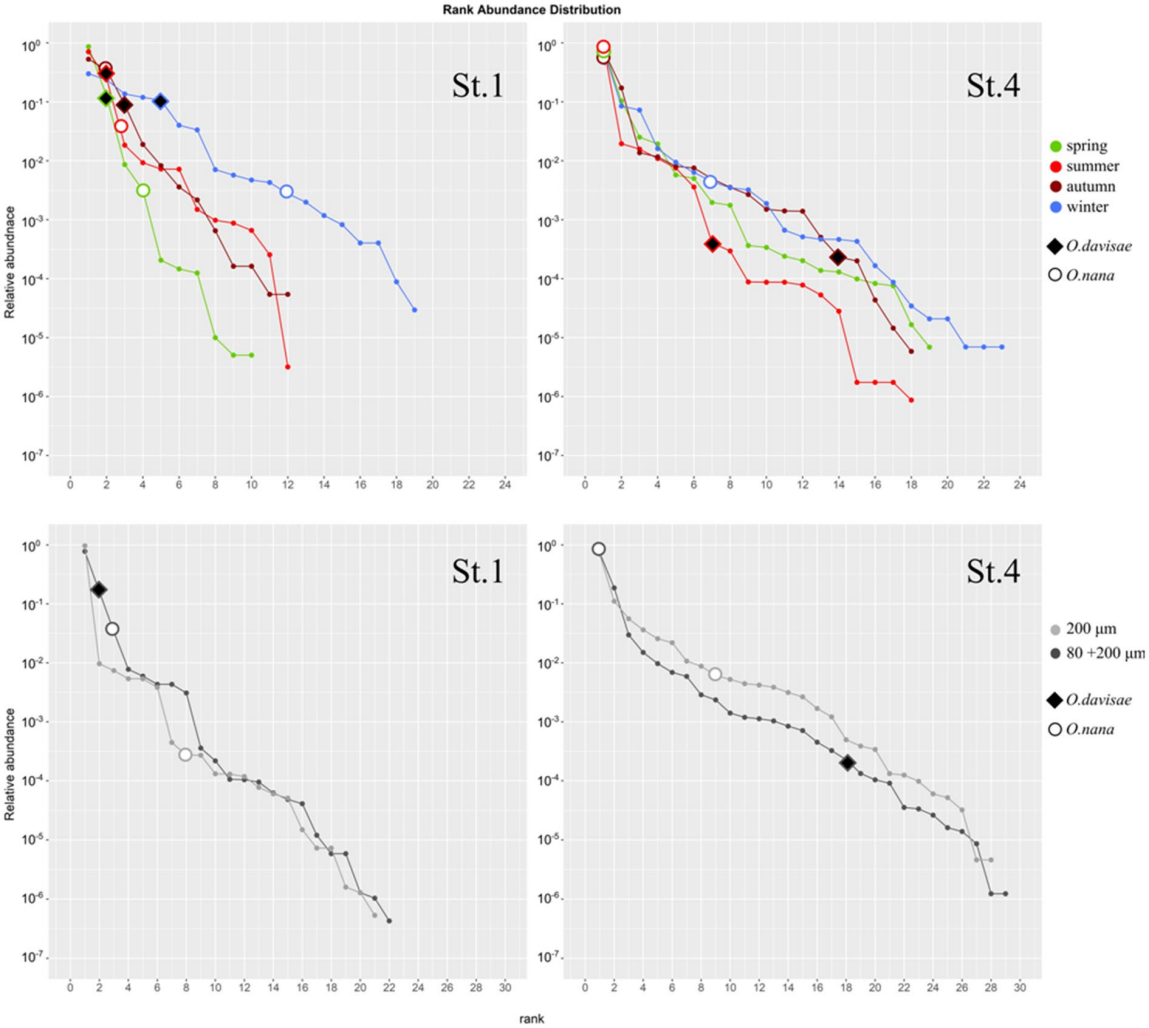
Upper panels: Synthetic Rank Abundance Distributions of the copepod community divided by seasons of St.1 and St.4 obtained by merging the data sampled with 200 μm mesh net over the period 2014–2017 and the data of monthly sampling (12 months) with the 80 μm mesh (2016–2017). Lower panels: Synthetic Rank Abundance Distributions of the copepod community of St.1 and St.4 comparing data of 200 μm mesh size only and data of merged data of 80 μm and 200 μm over the period 2014–2017. The ranks of the *Oithona davisae* and *Oithona nana* are highlighted.

The overall copepod communities (200 + 80 μm) were characterized by different number of species and evenness: St.1 is characterized by lower number of species, with a total of 22 species of copepods respect to 29 species detected at St. 4, and by a minor evenness highlighted by a steeper curve compared to St.4 (Fig. 5). The RAD curve of St.1 showed generally that *O. davisae* and *O. nana* are at the second and third rank place, respectively, within a community strongly dominated by *A. tonsa* that accounts for about 77% of the total density. At St.4, *O. nana* is at the first species rank, representing just over 70% of total community, and *O. davisae* at the eighteenth rank (Fig. 5). The community in St. 4 was characterized by a higher presence of marine species, both in terms of density and number of species, like *A. clausi, P. parvus, O. similis* and *Centropages ponticus.* The seasonal RADs of St.1 show a dominance of the copepod *A. tonsa* from spring to autumn, with relative abundances of about 86% and 70% in spring and summer (max abundance 13,293 and 10,015 ind/m^3^, respectively) and slightly higher than 50% in autumn. *Oithona davisae* is at second rank place in spring and summer, while in the same seasons *O. nana* is at fourth rank place preceded by *Acartia margalefi* in spring and at third rank place in summer. During winter, the community is characterized by higher species numbers and higher degrees of evenness compared to previous seasons. In this season, the copepod abundance is very low: the two species of Acartidae, *A. tonsa* and *A. clausi,* represented more than 50% of the copepod community (max abundances 170 and 166 ind/m^3^, respectively), and *O. davisae* is at the fifth rank place whereas *O. nana* at twelfth. At St.4, *O. nana* is dominant in spring, summer and autumn, with percentage of 83%, 94% and 77% respectively, while in winter is at the seventh rank place, within a community dominated by *A. clausi* (79%, max abundance 12,178 ind/m^3^). *Oithona davisae* was not ranked in winter and spring and resulted at seventh and fourteenth rank place in summer and autumn, respectively. Differently to St.1, where a trend of increasing diversity and evenness from spring to winter was observed, at St.4 the diversity was similar from spring to autumn and a little higher during winter, while no evident trend of increasing evenness was observed (Fig. 5).

In order to compare the results that would be obtained when using a zooplankton net with a mesh size of 200 μm, RAD curves based on this data only were computed. The shape of the RADs curves for St.1 and St.4 did not vary appreciably, but the rank of the two oithonid species changed significantly: *O. davisae* was not ranked, as it was not detected, both at St.1 and St.4, while the abundance rank of *O. nana* was only the ninth at St.4 and the eighth at St.1 (Fig. 5).

### Niche apportionment models

**Figure 6:**
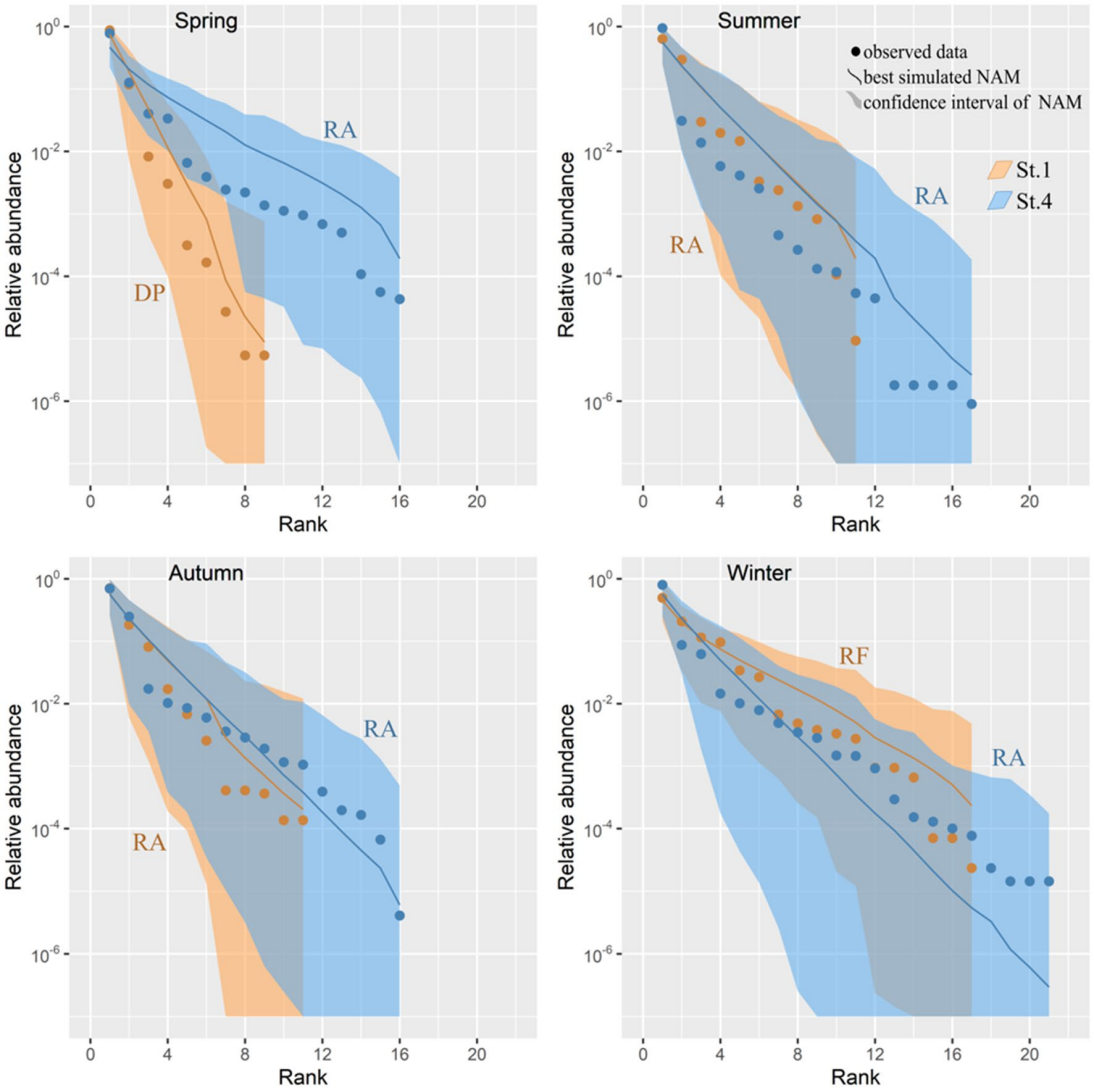
Best fitting Niche Apportionment Models for different seasons at St.1 and St.4.

Six Niche Apportionment Models (NAM) on the RADs of the copepod community for St.1 and St.4 in different seasons were tested. While for St.4, the differences in the fitting of apportionment models between seasons are minor, at St.1 the best fitting scheme differs between seasons (Table 3). This reflects, for St.1, an increasing trend in the evenness of the community from spring to winter: in spring, the best fitting model was the Dominance Preemption, characterized by a steeper RAD curve (Fig. 6). This model predicts that the first species that invades a resource space always occupies more than half of the available niche letting the remaining portion to be exploited by a second species in the same way and so on. Therefore, this species will be dominant on all the remaining species, resulting in a strong dominance structure. In summer and autumn, the best fitting model for both stations was the Random Assortment model (Fig. 6), which refers to a situation where the niche space is randomly divided among simultaneously or sequentially colonizing species. Thus, the abundances of different species vary independently of each other or, in other words, the species appear to be ecologically identical according to the neutral theory of biodiversity. This could arise both as a result of a mismatch between abundance and niche size or as dynamic non-hierarchical niche partitioning in a highly variable environment^45^. During winter, the best fit was for the Random Fraction model, with a RAD curve with a flatter slope than the previous ones, indicating a higher degree of evenness within the copepod community in this season (Fig. 6). This model is based on the assumption that sequentially colonizing species in an assemblage have the same probability of niche occupancy so that each new species carves out a random portion of the available niche space^44,46,52^. In this case, the coexistence mechanism during winter, characterized by a more diverse community, could rely on the development of independent population dynamics with random exponential growths under a variety of environmental limiting factors, as in Preston’s lognormal distributions. At St.4, the best fitting was for Random Assortment model all year round, but also a weak signal for Random Fraction was detected in spring, autumn and winter (Table 3). None of the schemes MacArthur Fraction and Dominance Decay exhibited a detectable fit.

**Table 3:** Testing of niche apportionment models at St.1 and St.4 in different seasons. Best fitting values are in bold. The models are in order of increasing evenness from left to right (DP: Dominance Preemption, RA: Random Assortment, RF: Random Fraction, PF: Power Fraction, MF: MacArthur Fraction, DD: Dominance Decay). Symbol “−” means no fit to data (*p* < 0.001).

Station	Season	DP	RA	RF	PF	MF	DD
St.1	Spring	**0.428**	0.009	–	–	–	–
	Summer	0.006	**0.681**	0.015	–	–	–
	Autumn	0.013	**0.653**	0.124	0.009	–	–
	Winter	−	0.066	**0.763**	0.056	–	–
St.4	Spring	−	**0.097**	0.025	–	–	–
	Summer	0.002	**0.134**	0.001	–	–	–
	Autumn	0.001	**0.282**	0.021	0.001	–	–
	Winter	−	**0.055**	0.021	–	–	–

## Discussion

The increasing anthropogenic activities, including maritime transport and aquaculture, enhance the spread of NIS at a global level^53,54^, potentially increasing the local biodiversity or endangering local communities. Climate change and the resulting increase in seawater temperature, especially in Mediterranean, as a marginal sea, and particularly in Northern Adriatic^55,56^, favour the spread of thermophilic invasive species. Alien species that occupy trophic niches, similar or close to those of native species, can alter the balance of the latter and lead to a significant reduction in their number^57,58^. The invasive copepod *O. davisae*, known to have potentially a negative impact on the local copepod community, is often reported among the dominant species within the copepod community, both in native and introduced areas^17,23,59^. In the past, the genus *Oithona* has been probably often underestimated since the coastal species are less than 1 mm in body length and the use of conventional mesozooplankton nets (200 μm mesh size) fails to quantitatively catch these small cyclopoid species, resulting therefore unsuitable to characterize their real abundance^12,40^. With respect to previous works [e.g. ^9^], the present study adopts a net equipped with a smaller mesh size, allowing to detect the presence of the species with greater precision both in terms of quantity and distribution area. The present study reports the first description of the spatial and temporal distribution of the non-indigenous cyclopoid copepod *O. davisae* in the VL, after its first observation in 2014^9^, including details regarding potential invasion patterns and the niche relationships with the autochthonous congener *O. nana* and the overall copepod community.

In this study, *O. davisae* revealed patterns of presence and abundances related to the seasonality, with highest densities in the warm period and very low abundances in the cold season. These data confirm the thermophilic character that this nearshore estuarine species exhibits not only in native areas but also where it is a recently-introduced player^20,60^. In the VL, the *O. davisae* population began to increase in May, when water temperature exceeded the values of 18–20 °C, threshold at which the population densities start to actively increase^61^. The maximum abundances in the VL were reached in June and July, the same months in which^18^ reported the highest densities of *O. davisae* in Lake Faro and Ganzirri (Strait of Messina, Italy), although with 20-fold greater than those observed in the VL. From August onward, the population exhibited a decrease in abundance. This trend could be also related, as reported for other areas^17,61^, to direct jellyfish predation. In our case by the invasive comb jelly *Mnemiopsis leidyi*, which started to become very abundant in the VL from August 2016 (personal observation). We do not know if *Mnemiopsis*, which is a generalist predator, is the main predator of *O. davisae* in VL, but we hypothesize that the decrease of *O. davisae* in August and September could also be due to the massive presence of *Mnemiopsis*. According to available literature, in the native areas and in those where it has been introduced, *O. davisae* presents a second peak of abundance in late summer/early autumn^20,59–61^. Conversely, this has not been observed in the VL where they actually exhibited very low densities in the same period, also depending on the sudden drop of water temperature and low chlorophyll *a* values. In winter, when the water temperature further decreased down to 1.2 °C, the species resulted almost absent, or it was not detected due to very low abundances. In the cold period, it is known that pre-fertilized *O. davisae* females reduce motion and respiratory activity by holding the sperms in the spermatheca until spring^61,62^. Therefore, these survival strategies may explain how the species copes with these extreme conditions despite its thermophilic characteristics. However, as mentioned above, in the VL the maximum abundances of *O. davisae* are not comparable with those reached in the native areas^17^, or in the Black Sea^63^ and in the Lakes of Faro and Ganzirri^18^. Another hypothesis regarding such relevant differences in abundance, could be related to the qualitative difference in food resources between VL and the aforementioned areas. The dominance of *O. davisae*, both in the Tokyo Bay and in Black Sea, was related to a shift from a diatom-based phytoplankton community towards one with prevalence of small flagellates^63,64^. *Oithona davisae* is, in fact, an ambush feeder that prefers motile preys like flagellates, ciliates and dinoflagellates^13,65,66^. Several authors reported these feeding preferences, both in laboratory experiments and in natural conditions, confirming that diatoms are not part of the diet of this species^67–69^. The second hypothesis explaining the low density of *O. davisae* in VL could therefore take into account the availability of suitable food. As described by^70^, the phytoplankton seasonal cycle in the VL shows a temporal distribution pattern which justifies the presence of *O. davisae* especially in certain areas and at certain times of the year: diatoms are in fact the dominant group all year round but, after their spring bloom, going toward summer, also cryptophyceans, dinoflagellates and euglenophyceans, appreciated food by *O. davisae*, appear in the phytoplankton community. This is consistent with our data and explains the high abundance of *O. davisae* only in June/July. This feeding preference may also explain the spatial distribution of *O. davisae* as it shows its highest densities especially at St.1 one of the most eutrophic areas in the lagoon^71,72^, where cryptophyceans, dinoflagellates and euglenophyceans were found to be abundant during the warm season^70,73^.

*O. davisae*, as most copepods, is able to form dense populations in short time^17^ and it can have a strong impact on the ecosystem structure and functioning especially following an ecological crash. In fact, in 1992, the copepod *A. tonsa* found the ideal conditions for its development and settlement during a critical event of hypertrophy followed by intense anoxia in the VL. In the case of the Black Sea, the successful introduction and later establishment of *O. davisae* has been preconditioned by the complete disappearance of *O. nana,* previously caused by the invasion of *M. leidyi,* enabling *O. davisae* to occupy this empty niche^74^, and later, by the reduced predation pressure of *M. leidyi* on zooplankton due to the predation of *Beroe ovata* on the ctenophore. Similarly, in Lakes Faro and Ganzirri (Sicily), the occasional remixing of anoxic bottom waters rich in hydrogen sulfide, in conjunction with high temperatures during summer (> 32 °C^75^), caused the complete disappearance of zooplankton (Zagami, pers. comm). The temporarily underexploited niche, in the Lakes Faro and Ganzirri, could have then provided an opportunity for the successful introduction and establishment of *O. davisae*. In the VL, at present, there have been no documented ecological crashes and the possible role of grazing pressure on zooplankton and on *O. davisae* in particular is still undocumented, though under study. However, rather than a niche left possibly empty, predation is expected to be the factor controlling the spread and dominance of zooplankton species in the Lagoon.

In addition to niche employment, replacement through a competitive exclusion mechanism was also observed^18,26^. This latter phenomenon has been explained assuming that the ecological traits of *O. davisae* may confer competitive advantage over *O. nana* or other species, like *A. tonsa* in the Black Sea^59^. At the moment, such species replacement has not yet occurred in the VL probably because of the extreme variety of habitats and environmental niches offered by this environment^76^ allowing a balanced coexistence with *O. nana* and in general with the resident copepod community. In fact, according to our data, the population of *O. davisae* has not exceeded, in terms of abundance, those of *O. nana* except in the internal and more eutrophic station (St.1) in the hot period.

Based on the different distribution gradients and the two target stations, one characterized by higher degrees of marine influence (St.4) and the other by greater degrees of confinement (St.1), in the present work the comparison of the synthetic RAD curves allows to define an updated and more reliable picture of the species rank of *O. davisae*, including its role with respect to the autochthonous congeneric *O. nana* and other copepod species like *A. tonsa*, which is dominant in the same areas where *O. davisae* has also currently settled^36^.

At St.1, the inner station, the copepod community showed a lower species richness and evenness compared to St.4, the more marine station close to the inlet, confirming the difference between these two areas in term of habitat features. The more restrictive environmental conditions of the inner station (St.1) trigger in fact a selection among competing species, regulated by environmental factors, limiting the species richness and favouring the most rapidly growing and tolerant ones. The inlet station (St.4) conversely, due to the marine influence and the resulting mixing, with more stable environmental conditions, maintains a more even species richness, regulated by interspecific competition. At St.1, *O. davisae* and *O. nana* were, together with *A. tonsa*, the three most abundant species and ranked at the second and third places respectively, while at St.4, *O. nana* was at the first and *O. davisae* at the eighteenth rank place. Also, with respect to the calanoid copepod *A. tonsa*, which shares the same distribution pattern of *O. davisae*, these species seem to coexist or perhaps *O. davisae* is still controlled in terms of abundances by the presence of *A. tonsa*. Both species are opportunistic and benefit from more unstable environmental conditions. An analysis of the interannual abundance of copepods in the Sevastopol bay showed a reduction in the abundances of the previous invader *A. tonsa* after the arrival of *O. davisae*^59^. Nonetheless, in the VL, the establishment of *O. davisae* does not seem to have caused a significant reduction in the abundances of *A. tonsa,* probably because to low abundances of *O. davisae* until now. From its first report in the VL to date, *O. davisae* has not dominated over *A. tonsa*, but only the continued monitoring of the evolution of the abundance and distribution dynamics of these species will allow to define their respective roles and/or dominance within the trophic lagoon network over time.

To support inferences on whether the mechanisms that regulate the resource allocation among the copepod guilds could favor or not the diffusion of *O. davisae* in the Lagoon of Venice, we tested the performances of several model of niche apportionment (NAMs) on the seasonal copepod abundances in the two already mentioned areas of the Lagoon (St.1 and St.4). Niche apportionment models support the investigation of the mechanisms of apportionment of the resource pools in different habitats. When a niche contender, generally a non-indigenous species (NIS), is introduced into a new environment, the scenarios that this will trigger can be various. At St.1, the picture highlighted by the NAMs well describes the changes in the resource partitioning mechanisms. Starting from spring, the low-richness community is strongly dominated by *A. tonsa* and *O. davisae,* as major competitors in pre-emptying the resource pool and subtracting niche space to the less abundant species. Then the picture gradually evolves in Summer and Autumn, into a situation in which the available niches are unsaturated, as the high variability and the rapid changes in environmental conditions do not allow the establishment of strong competition mechanisms between species. The mechanism of apportionment randomly assigns the niche space to species irrespective to their abundances, which vary independently from each other. Finally, in Winter more stable environmental conditions and low productivity lead instead to the establishment of competition mechanisms without strong dominance of one or few species in a relatively richer and more even community. *O. davisae* could then be favoured, in the warm season at St.1, not only by suitable local habitat conditions, both trophic and physical, but also by the established mechanisms that regulate the resources allocation. The NAMs of St.4 showed a relatively constant situation throughout the year, with the best fitting for Random Assortment model, but also with a weak signal for Random Fraction. In our opinion, this could be the effect of the intrinsic characteristics of the station at the inlet, where water and organisms of marine and estuarine origin periodically mix: these results in a blended community where both randomness and competition mechanisms determine the relative ranks. As a consequence, the trophic and physical local habitat features, disliked by *O. davisae*, may limit its distribution to the more confined and eutrophic area and this is reflected by the nice partitioning mechanisms acting on the copepod guilds at this station.

To date, *O. davisae* is well-established in the VL, but we do not know yet if the species has reached its growing plateau. Mechanisms of competitive exclusion with *O. nana*, as reported for other areas, have not been observed in the VL. In our opinion the most credited hypothesis is that there is not overlapping between the preferred habitat by the two congeneric species and the extreme habitat variability leads to coexistence. However, continuing a constant monitoring with the approach of combined net mesh size will allow to get a more realistic assessment of the mesozooplankton community, thus observing the future evolution of the populations of the two species. This updated picture of the copepod community, with a view on the arrival of a new species, also highlights and confirms that the long-term monitoring programmes of zooplankton are an essential tool for sustainable development and environmental management.

## Supplementary Information

Supplementary Information
