# Supplementary material for: The non-indigenous Oithona davisae in a Mediterranean transitional environment: coexistence patterns with competing species
Source: Sci Rep. 2021 Apr 16;11:8341. doi: 10.1038/s41598-021-87662-5 (PMC8052375; doi:10.1038/s41598-021-87662-5)
Supplement: Supplementary file 1 — Supplementary Information [file 41598_2021_87662_MOESM1_ESM.pdf]

Supplementary information to:

“The non-indigenous *Oithona davisae* in a Mediterranean transitional environment: coexistence patterns with competing species”

Marco Pansera[1], Elisa Camatti[1*], Anna Schroeder[1,2], Giacomo Zagami[3], Alessandro Bergamasco[1]

[1] *National Research Council, Institute of Marine Sciences (CNR ISMAR) Venice, Arsenale Tesa 104, Castello 2737/F, 30122, Venice, Italy*
[2] *University of Trieste, Faculty of Environmental Life Sciences, Via Licio Giorgieri 5, 34127 Trieste, Italy*
[3] *Department of Chemical, Biological, Pharmaceutical and Environmental Sciences, University of Messina, Viale F. Stagno d'Alcontres, 31, S. Agata, 98166, Messina, Italy*
* correspondence: elisa.camatti@ismar.cnr.it

The method used to merge the *O. davisae* and *O. nana* data coming from the monthly 80 µm (2016-2017) dataset within the seasonal 200 µm (2014-2017) dataset of the overall copepod community is described below. The 200 µm data were not interpolated. The interpolated 80 µm data were merged with the 200 µm data by replacing only the 200 µm value of *O. davisae* and *O. nana* with the interpolated (80 µm) value of *O. davisae* and *O. nana.*
Samples with 80 µm net: Available 12 samples evenly spaced in time (i.e. one sample per month) during 2016-2017 and counts regarding *O. davisae* and *O. nana* species

		**St. 1 (S. Giuliano)**		**St. 4 (Lido)**	
**Date**	**DOY**	*O. davisae*	*O. nana*	*O. davisae*	*O. nana*
19/01/17	18	0	6	0	297
20/02/17	49	56	0	0	35
24/03/17	83	0	3	0	76
23/04/17	112	53	3	0	508
04/05/17	123	264	23	0	1072
19/06/17	168	6490	126	0	20617
21/07/17	200	2338	176	15	28370
24/08/16	233	53	147	18	5374
23/09/16	262	352	15	9	4699
28/10/16	297	123	2226	0	8399
23/11/16	322	32	167	3	1043
07/12/16	336	9	9	3	405

Supplementary Table S1

Samples with 200 µm net: Available seasonal samples during 2014-2017 and counts regarding all the copepod species. Due to coarse mesh size, *O. davisae* is absent (no catches at all) and *O. nana* is scarce

		**St. 1 (S. Giuliano)**			**St. 4 (Lido)**		
**Season**	**Date**	**DOY**	*O. davisae*	*O. nana*	**DOY**	*O. davisae*	*O. nana*
Winter	11/02/2015	41	0	0	41	0	5
Winter	20/02/2017	51	0	0	51	0	45
Spring	29/04/2016	119	0	0	119	0	22
Spring	21/05/2014	-	-	-	140	0	30
Spring	27/05/2015	146	0	0	146	0	10
Summer	10/07/2014	-	-	-	190	0	10
Summer	14/07/2016	195	0	1	195	0	22
Summer	08/08/2014	219	0	8	-	-	-
Autumn	01/10/2014	273	0	0	-	-	-
Autumn	21/10/2014	-	-	-	293	0	4
Autumn	13/11/2016	317	0	2	317	0	39

Supplementary Table S2

Problem: How to detail the presence/absence and relative abundances of the two species (*O. davisae* and *O. nana*) that are clearly underestimated through the sampling with 200 µm net within the picture of the overall copepod community in each station and possibly in each seasonal/environmental condition.

Adopted solution: We approached the issue by assuming as "possible" the yearly trend of *Oithona* species coming from the samplings with 80 µm net. The linear interpolation of the expected abundances in specific calendar days (DOY) made possible their merging with the specimen counts of the 200 µm samplings.

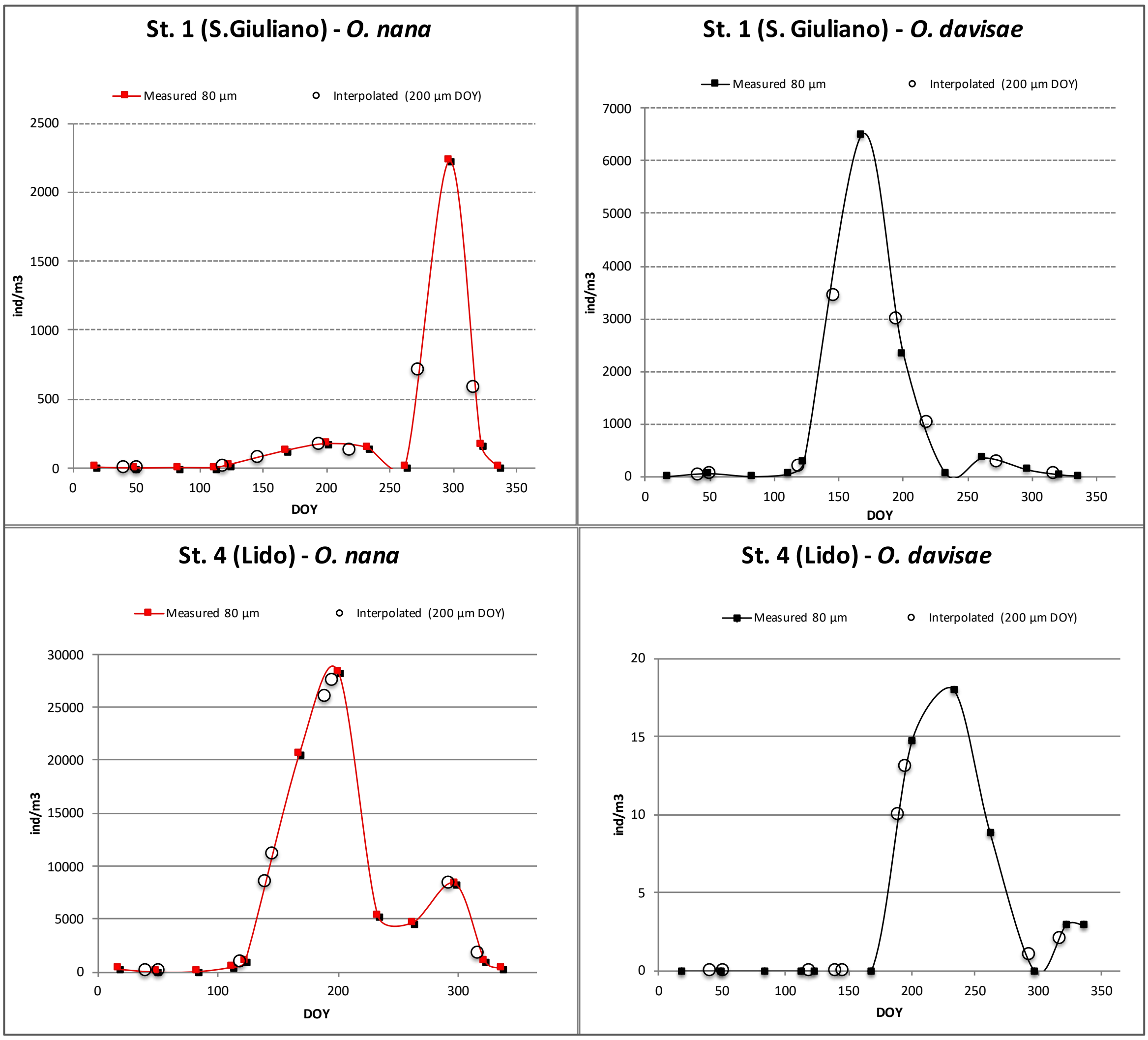

Supplementary Figure S1

The yearly abundance trends coming from the 80 µm samples are shown in Figure S1 in continuous line for the two species at the stations San Giuliano (St.1) and Lido (St.4). Linearly interpolated abundances in the calendar days (DOY) when 200 µm samples exist are shown (empty bullets).

	St. 1 (S. Giuliano)			St. 4 (Lido)		
**Season**	**DOY**	*O. davisae*	*O. nana*	**DOY**	*O. davisae*	*O. nana*
Winter	41	23	2	41	0	50
Winter	51	54	0	51	0	40
Spring	119	187	16	119	0	850
Spring	-	-	-	140	0	8456
Spring	146	3446	76	146	0	11062
Summer	195	2987	168	190	10	25947
Summer	219	1022	130	195	13	27500
Autumn	273	280	710	293	1	8300
Autumn	317	50	579	317	2	1700

Supplementary Table S3: Linearly interpolated 80 µm *O. davisae* and *O. nana* abundances

These interpolated abundance values (supplementary Table S1) can be merged with the abundances of the other copepod species collected with the 200 µm mesh to have the overall picture of the community (without underestimation or with reduced underestimation) for further processing (e.g. NAM).

Sampling dates of the 200 µm dataset

**Seasonals samplings (2014-2017)**	
Spring	21/05/14
Summer	10/07/14
Summer	08/08/14
Autumn	01/10/14
Autumn	21/10/14
Winter	11/02/15
Spring	27/05/15
Spring	29/04/16
Summer	14/07/16
Autumn	13/11/16
Winter	20/02/17

Supplementary Table S4: Sampling for the 200 µm dataset 2014-17
